# Palmer’s Artifical Limbs

**Published:** 1848-01

**Authors:** 


To the Editor of the Boston Medical and Surgical Journal

*Palmer's Artifical Limbs.*—My Dear Sir,—In compliance with your request and of many others of the medical faculty, I have written this short article, which I send for publication in your Journal, concerning my patent artificial limb. I will not attempt to give an elaborate account of all the minutiæ of its construction, but simply a *general* idea of its novelty, utility and *modus operandi.* It would be quite impossible, in this short sketch, to give your readers anything like a definite idea of the peculiar action of the various parts about the articulations, or the new and improved modes of constructing and attaching the springs, tendons &c., which give life-like action to the substitute; and, as it is not the manner of arranging, but the result produced by the improved principles combining and acting in the substitute which will more particularly gratify the reader, I shall aim to give an idea of the leg in operation, and make some suggestions to the profession concerning amputations, as my success depends somewhat upon the place of amputation. Many have visited me for relief, whom, to relieve, was almost to *create*, in consequence of unskilful amputation. It may not be uninteresting to your readers to know that this invention of the artificial leg, is the result of much investigation, years of incessant thought, and days and nights of unmitigated toil, in consequence of the loss of one of the inventor's legs, which was ground off in a bark mill, when he was eleven years of age. All the most approved artificial legs, including the valuable one of Dr. Potts, of London, know as the "Anglesey Leg," I had worn previous to completing my invention, and I tested thoroughly my own improvement, and secured a patent for the same, before venturing to expose it to the gaze of a scrutinizing public. In fact, I only thought, originally, to relieve my own suffering by the production of my own hands; but having exhibited the limb thus constructed, I was called before the public, and a generous American public has fully appreciated the worth of the invention. My unfortunate fellow beings, who, like me, had travelled long on crutches o'er earth's crags, without hope of relief, have arisen, as one has said, "in joint and limb complete," and my fondest hopes are more than crowned with success. I trust you will pardon the momentary digression in which I have indulged, and I will now return to the subject of my letter.

As much depends upon a skilful amputation, and as various erroneous ideas have been conceived by many surgeons with regard to the *best stump* to which to fit an artificial limb, permit me to remark that *generally* it is desirable to have the stump left as long as it can be with safety. The question may be asked, "is not this now the case"? I answer, no. I have now with me a gentleman, whose leg was amputated very near the knee-joint, which might just as well have been taken off in the most desirable place for fitting an artificial one, so as to give the most *complete* and *perfect* use of the natural knee; but the patient was told by the surgeon that 'twas in vain to think of applying a leg below the knee, so as to give the use of the natural joint, without great difficulty in supporting the weight, and that it is was preferable to flex the knee, and pressing the thigh into a socket—"*push along*," as *Hood* would say, "thump, lump—lump, thump," upon the uncouth stub leg. Prodigiously absurd is such an idea; and even now in this case, with this very short stump, I shall give the prtient a very tolerable use of the natural knee, as I have done in several similar instances previously. But how admirably he might have walked, if the amputation had been performed a few inches lower—and the *injury* was very near the ankle, so that the leg *might* have been removed very low down. I always use the natural knee, if flexible, if there remains two and one half inches of the limb below the joint. If the amputation be below the knee, and the articulation flexed and *stiff*, the leg remaining at right angles with the thigh, or nearly so, with my apparatus I give perfect use of an artificial knee-joint, without elongating the thigh or increasing its circumference enough to be perceptible. In no case does any pressure come upon the end of the stump, so that, however tender it may be about the place of amputation, as the bearing comes upon the circumference of the limb above. the weight is supported with perfect ease. A stump from six to ten inches in length *below* the knee gives the best chance for fitting a false limb. If the thigh is to be amputated, let the operation be also performed as low as practicable, though it should not be within three inches of the knee. I make these suggestions, believing them to be important, as many limbs are amputated at random, or at least without any reference to the subject now under consideration; and I am certain that you would not deem them unimportant, if you were in the habit of fitting artificial limbs, as I am, to all the various kinds of sumps. Permit me, however, to remark, that in all cases of amputation, if there remain two inches of the thigh, a limb may be fitted so as to be far preferable to a crutch, and enable the wearer to walk with facility, and engage in any active employment. A gentleman for whom I arranged a limb last winter, whose thigh was amputated near the body, informs me that he labored upon a farm the past summer, and could perform as much labor and command as high wages as any common farmer, and that he had travelled on foot four miles in sixty-five minutes with his new leg. In all cases where any of the natural articulations are either gone or inflexible, I give the patient perfect use of artificial ones to supply the deficiency thereof.

The principles upon which my patent leg is construted, are such that a complete likeness of the anatomical limb is given, and whether dressed or undressed there appears no excrescence, vacuum or unnatural depression in the contour. About the articulations, also, the same symmetry of proportion is observed as in natural joints. The springs and tendons in the artificial limb which perform the functions of the *tendo-Achillis*, flexor and extensor muscles, &c.

are durable, and as they are all in the interior of the limb, they are not subject to accident, and do not wear the clothing covering the leg, or injure the beauty and finish of the same. I have compounded a cement which I use in the finish of the exterior, which is impervious to water, and which fastens the outside covering, or cuticle, indissolubly, and gives a beauty of color and finish, rivalled only by that of the human limb. B. Frank. Palmer.

Meredith Bridge, N. H., Nov. 24th, 1847.

[*From the Boston Med. and Surg. Jour.*

